# Detailed genetic and clinical analysis of a novel *de novo* variant in *HPRT1*: Case report of a female patient from Saudi Arabia with Lesch–Nyhan syndrome

**DOI:** 10.3389/fgene.2022.1044936

**Published:** 2023-01-26

**Authors:** Albandary AlBakheet, Hanan AlQudairy, Joud Alkhalifah, Sheikhah Almoaily, Namik Kaya, Zuhair Rahbeeni

**Affiliations:** ^1^ Department of Translational Genomics, Center for Genomic Medicine, King Faisal Specialist Hospital and Research Centre (KFSHRC), Riyadh, Saudi Arabia; ^2^ College of Medicine, King Saud University, Riyadh, Saudi Arabia; ^3^ College of Medicine, AlFaisal University, Riyadh, Saudi Arabia; ^4^ Department of Medical Genetics, Center for Genomic Medicine, KFSHRC, Riyadh, Saudi Arabia

**Keywords:** *de novo*, *HPRT1*, X-inactivation assay, RTPCR, Lesch–Nyhan syndrome

## Abstract

**Background:** Hypoxanthine-guanine phosphoribosyltransferase (HPRT1) deficiency is an inborn error of purine metabolism responsible for Lesch–Nyhan syndrome (LNS). The disease is inherited in an X-linked recessive manner and predominantly affects male individuals. Female individuals can carry a mutation as heterozygotes, but typically, they are asymptomatic because of the random inactivation of the affected allele. Nevertheless, although rare, heterozygote female individuals may manifest LNS with full characteristics. Herein, we describe a female patient from Saudi Arabia with LNS.

**Results:** The patient (a 4-year-old girl) presented with typical characteristics of the disease, which include global developmental delay, self-mutilation, hyperuricemia, hypotonia, speech delay, spasticity, and seizures. Her general biochemical laboratory results were normal except for high levels of uric acid. The abdominal MRI\MRS, mostly unremarkable, showed bilateral echogenic foci within the renal collecting system. Genetic testing (whole-exome sequencing, iterative variant filtering, segregation analysis, and Sanger sequencing) pointed a novel *de novo* frameshift variant in *HPRT1*. X-inactivation assay using HpaII showed the presence of a 100% skewed X chromosome carrying the affected allele. RT-PCR of the cDNA indicated complete loss of the expression of the normal allele.

**Conclusion:** Our study presents a female patient who has a severe case of LNSand found to be the 15th female patient with the disease in the world. The study emphasizethe need for a streamlined protocol that will help an early and accurate diagnosis of female LNS patients to avoid unnecessary interventions that lead to costly patient care.

## Introduction

Lesch–Nyhan syndrome (LNS; OMIM: 300322) is a rare neurogenetic disorder of purine metabolism caused by complete or severe deficiency of the enzyme HPRT1 (Seegmiller et al., 1967). The disease is transmitted in the X-linked recessive mode of inheritance and encountered in different populations including Arabs (Shaltiel et al., 1981; Torres and Puig, 2007; de Gemmis et al., 2010; Yamada et al., 2014; Nguyen et al., 2017; Cho et al., 2019; Li et al., 2022). Hyperuricemia, dystonia, cognitive deficits, and self-injurious behavior are among the reported characteristics of LNS (Lesch and Nyhan, 1964). Mutations in *HPRT1* which is located on the long arm of the X chromosome (Xq26.1), are the main genetic causes of LNS (Simpson et al., 1988; Davidson et al., 1991; Sculley et al., 1991; Torres et al., 2000). Heterozygote female individuals are usually phenotypically normal, and the HPRT1 activity in their hemolysate is commonly normal, whereas heterozygote male individuals are affected (Torres and Puig, 2007). However, rarely, female carriers can be symptomatic, and so far, only 14 female individuals with heterozygous mutations in *HPRT1* have been described with full biochemical and clinical manifestations of LNS (Hooft et al., 1968; Hara et al., 1982; Ogasawara et al., 1989; van Bogaert et al., 1992; Yukawa et al., 1992; Yamada et al., 1994; Aral et al., 1996; De Gregorio et al., 2000; Jinnah et al., 2006; Sebesta et al., 2008).

In this study, we report a novel *de novo* frameshift mutation that led to LNS in a female patient from Saudi Arabia. The HPRT1 deficiency was confirmed based on previously described functional studies (Hara et al., 1982; Ogasawara et al., 1989; van Bogaert et al., 1992; Yukawa et al., 1992; Aral et al., 1996; De Gregorio et al., 2000; Rinat et al., 2006).

## Materials and methods

### Case report

**FIGURE 1:**
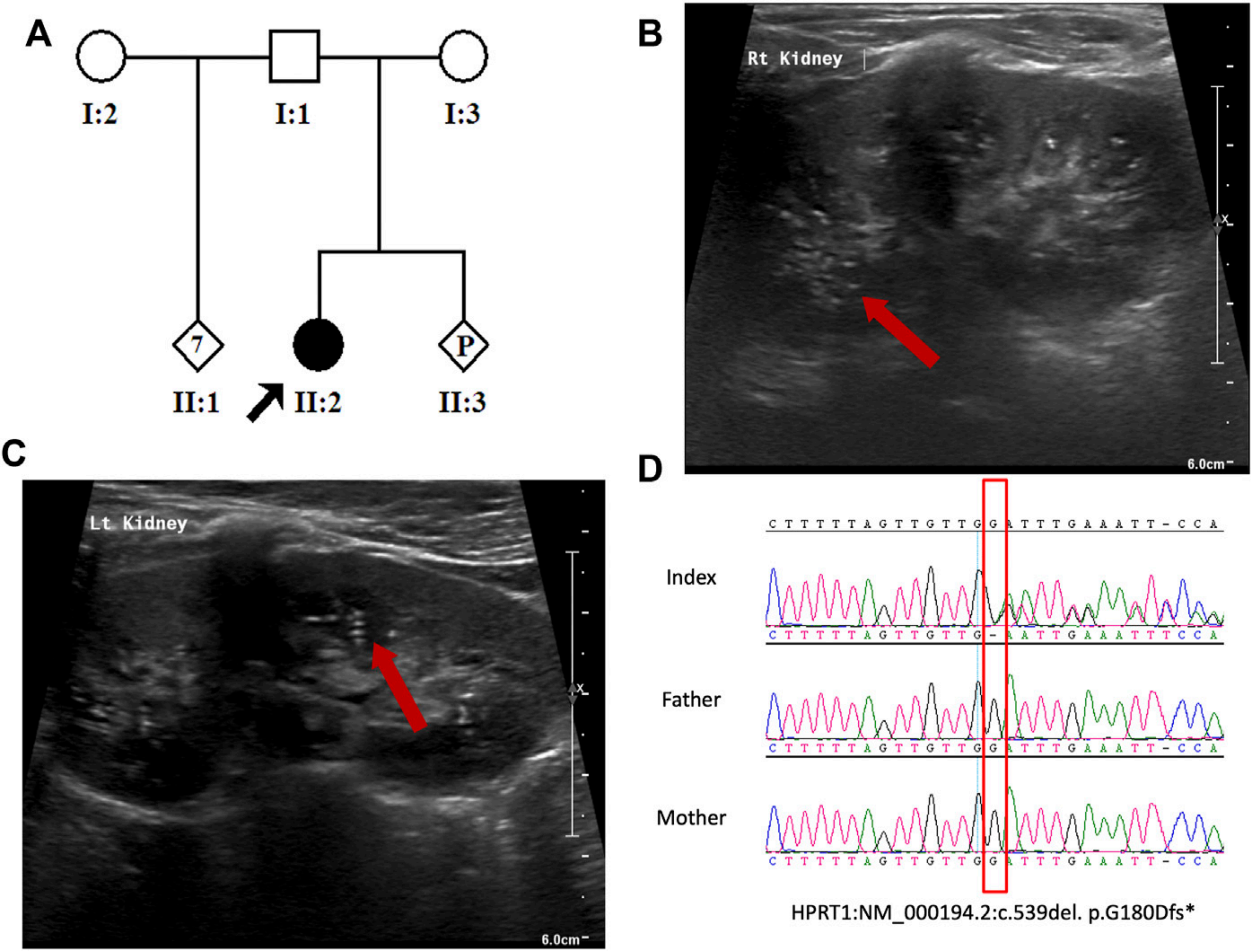
**(A)** Pedigree of the study family. **(B,C)** Abdomen US indicating bilateral echogenic foci within the renal collecting system. **(D)** Sanger sequence chromatogram showing the heterozygous *HPRT1* variant (NM_000194.2:c.539del. p. G180Dfs*10) in the patient and wild-type sequence in her parents.

A female patient from a nonconsanguineous Saudi family was recruited from a medical genetics clinic at the King Faisal Specialist Hospital and Research Centre (KFSHRC) (Figure 1A). Peripheral blood samples were collected into EDTA tubes from the affected girl and her parents after obtaining the signed informed consent approved by the institutional review board (KFSHRC Research Advisory Council, RAC#2120022). Skin biopsy collected from the affected individual was used for primary skin fibroblast culture.

### Molecular genetic analysis

Genomic DNA (gDNA) was isolated from blood using a Gentra^®^ Puregene DNA Purification Kit (Gentra Systems, Inc. Minneapolis, MN, US), according to the manufacturer’s instructions. Whole-exome sequencing (WES) was performed on the patient’s DNA, as described previously (Aldhalaan et al., 2021; Scala et al., 2022). To confirm the result, gDNA samples were amplified by PCR using HPRT1-specific primers (forward GTG​AAA​AGG​ACC​CCA​CGA​AG and reverse CAA​ATT​ATG​AGG​TGC​TGG​AAG​GA). The total RNA was extracted from the cultured fibroblasts using a QIAamp^®^ RNA Blood Mini Kit (QIAGEN^®^, Hilden, Germany). cDNA was synthesized using a High-Capacity cDNA Reverse Transcription Kit (Thermo Fisher Scientific Corp.). *HPRT1*-specific primers targeting the variant site were used for RT-PCR (forward CAA​AGA​TGG​TCA​AGG​TCG​CA and reverse ACA​GTT​TAG​GAA​TGC​AGC​AAC​T). Direct sequencing of PCR and RT-PCR products was performed on an ABI PRISM 3100 Genetic Analyzer (Thermo Fisher Scientific Corp.), according to the manufacturer’s recommendations. Quantitative RT-PCR experiments were performed by “quantitative SYBR green qPCR” assay (Thermo Fisher Scientific Corp.). *HPRT1* primers used for RT-PCR were utilized to quantify the level of mRNA. The experiments were performed on an ABI PRISM 7700 cycler (Thermo Fisher Scientific Corp.) using the PCR-efficiency-corrected -ΔΔCt method. The expression levels were normalized to *GAPDH* (FW: 5-TGC ACCACC AAC TGC TTA GC-3; REV: 5-GGC ATG GAC TGT GGT CAT GAG-3, GenBank NM_002046), as described previously (Livak and Schmittgen, 2001; Schmittgen, 2001). Variant frequency was obtained from the beta gnomAD browser and in-house database (n = 2,379).

### X-inactivation analysis

Analysis of the X-inactivation pattern was performed, as previously described (Torres and Puig, 2017). Briefly, the total genomic DNA was extracted from whole blood and fibroblast of the index case. The DNA was incubated with and without the *Hha*I restriction enzyme at 37°C for 16 h. Amplification of each sample was performed with the digested and undigested genomic DNA using FAM-labeled primers targeting the human androgen receptor (AR) (sense: 5′-TCC​AGA​ATC​TGT​TCC​AGA​GCG​TGC-3′ FAM; antisense: 5′-GCT​GTG​AAG​GTT​GCT​GTT​CCT​CAT-3′) to distinguish between maternally and paternally inherited alleles. The resulting products from the digested and undigested PCR were run on an ABI 3130 genetic analyzer and analyzed by GeneMapper 4.0 software (Thermo Fisher Scientific Corp.).

## Results

### Clinical features

The patient is a 4-year-old Saudi girl born to a nonconsanguineous couple after a full-term uneventful pregnancy *via* vaginal delivery (Figure 1A). Her mother is a 30-year-old Moroccan woman, and her father is a 42-year-old Saudi man who has seven unaffected children from his previous marriage. There was no family history of a similar condition. The patient’s prenatal and natal histories were all normal. At 2 years of age, she was referred to our institution for global developmental delay. According to her mother, she was neither rolling over nor able to sit, walk, or able to say any words. Based on the patient’s status, her developmental age was suggested to be 3–4 months. At 3 years of age, due to COVID-19 precautionary measures, she received a consultation over the phone. She developed self-mutilation and had an elevated serum uric acid level. After contacting her father, she was then admitted for lip surgery to assess her injuries at a local hospital. Molecular testing was utilized to assess her case. According to her WES results, a heterozygous variant in *HPRT1* was identified as the most plausible candidate matching her clinical phenotype. At 4 years of age, she displayed abnormal movement and was admitted electively to the hospital to investigate her state. During her hospitalization course, she was advised to see an occupational therapist to assess her self-mutilation, where she was offered a splint to help decrease her injuries. Her abnormal movements were then investigated by brain magnetic resonance imaging (MRI) performed under general anesthesia. MRI revealed no abnormality. A routine electroencephalogram (EEG) was performed for 20 min and showed continuous EEG recording with the normal anterior to posterior gradient. The EEG indicated mild non-specific cerebral dysfunction over the left temporal head region. Her abdomen ultrasound (US) showed a normal liver and spleen, but the urinary bladder was under-filled. Her kidney demonstrated a slight small right kidney for her age and bilateral echogenic foci within the renal collecting system (Figures 1B, C). These findings could represent uric acid deposition *versus* early nephrocalcinosis. There was no apparent shadowing that would indicate renal stones. She had an elevated serum uric acid level of 370 µmol\L. Her CBC and chemistry results were all within normal ranges (Table 1). Ophthalmologist consultation showed intermittent extropia. Allopurinol was given to the patient to control the uric acid level. The therapeutic modalities are merely supportive rather than curative, such as the use of wheelchair for mobility, protective masks, and elbow restraints to prevent self-injuries and reconstructive surgeries.

**TABLE 1:** Biochemical laboratory results of the patient.

Biochemical laboratory results		
Blood		Normal range
Uric acid (mmol/L)	403	60–240
Urea nitrogen (mmol/L)	5.6	1.5–6.8
Creatinine (mmol/L)	30	23–40
Sodium (mmol/L)	140	135–142
Potassium (mmol/L)	4.2	3.5–5
Chloride (mmol/L)	105	98–111
Glucose (mmol/L)	4.2	3.9–6.9

### Molecular genetics

**FIGURE 2:**
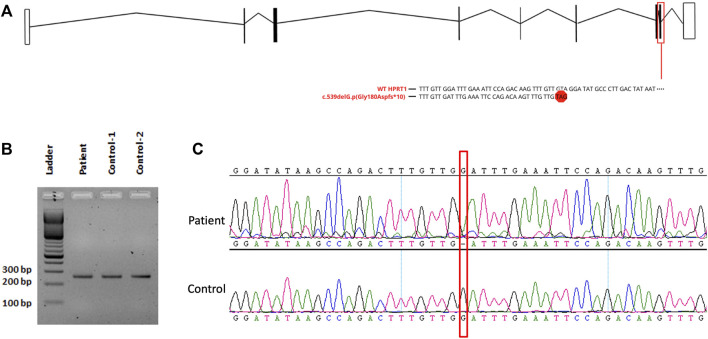
**(A)** Schematic drawing of HPRT1 transcript displaying the affected exon with the variant. **(B)** Analysis of RT-PCR on fibroblast-derived RNA, followed by 2% agarose gel electrophoresis. **(C)** Sanger sequencing for cDNA showing the presence of the mutant allele.

The result of WES analysis revealed a single conceivable candidate, a novel *de novo* heterozygote frameshift variant in exon 8 of *HPRT1* (NM_000194.2: c.539delG:p.Gly180Aspfs*10), which is neither found in gnomAD nor in in-house Saudi exomes (n = 2,379). The variant is in the highly conserved catalytic domain of HPRT1. Sanger sequencing of DNA demonstrated both normal and mutant *HPRT1* alleles in the affected female patient, while her parents did not have the mutant allele, indicating that the mutation is *de novo*. (Figure 1D). The variant was classified, according to the American College of Medical Genetics (ACMG) guidelines, as probably pathogenic with the criteria being PVS1 (strong evidence for cosegregation) and PM2 (absent gnomAD exomes). Sanger sequencing of the amplified genomic DNA (Figure 1D) revealed one base deletion in the patient. Such a change presumably leads to a premature stopcodon and causes a severely truncated protein (Figure 2A).

**FIGURE 3:**
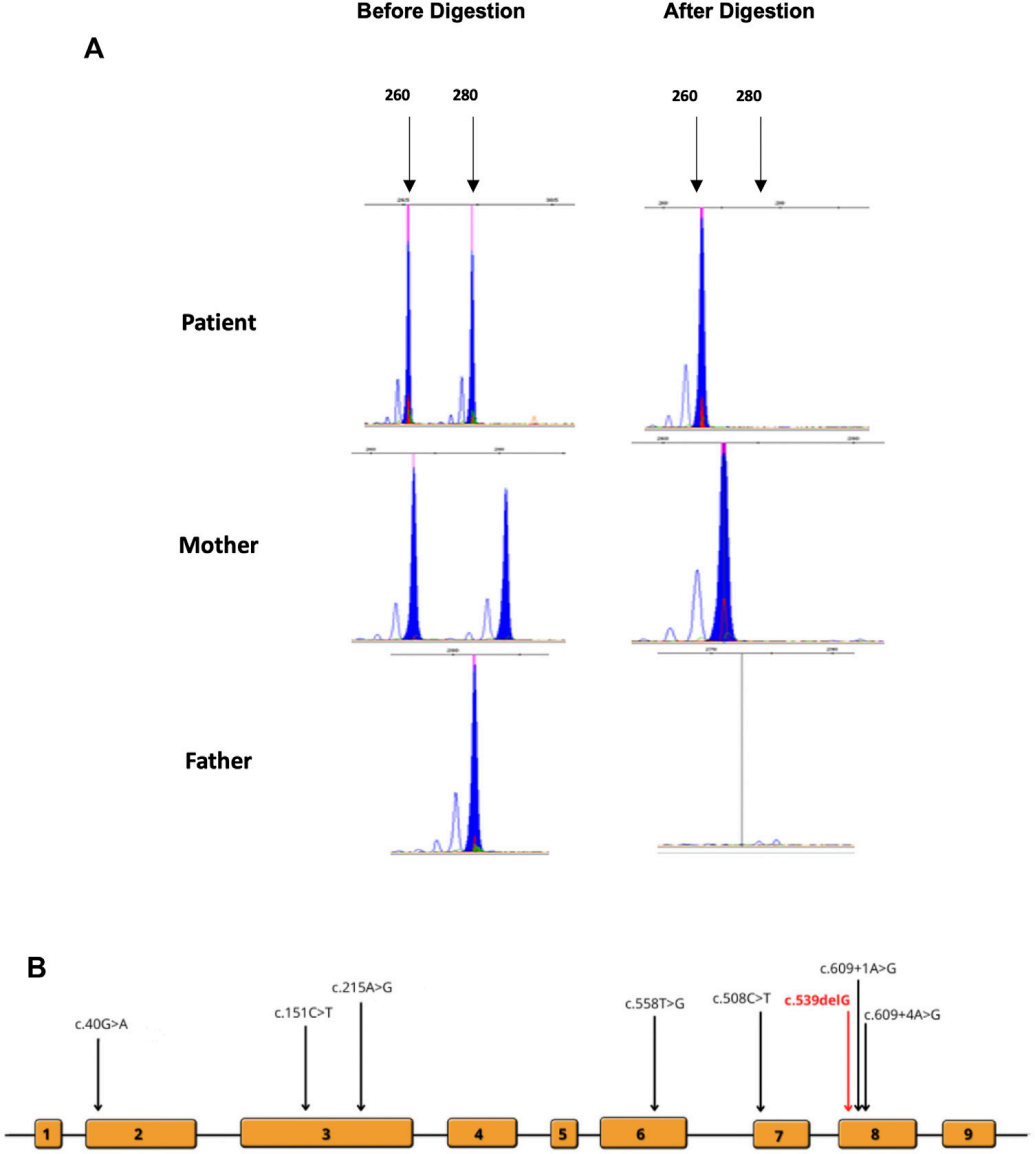
**(A)** Analysis of X-inactivation as signified by the AR locus. DNA was amplified before and after *HhaI* digestion. **(B)** Locations of the previously reported mutations are indicated on the schematic diagram of the gene and exons.

RT-PCR analysis (Figure 2B) did not show any significant size difference on an agarose gel (2%). However, Sanger sequencing of the cDNA prepared from the total RNA extracted from the cultured fibroblast of the affected female patient revealed the complete absence of the mRNA of the normal allele and showed only the transcription of the abnormal allele of *HPRT1* (Figure 2C). The absence of a normal allele implicates the presence of non-random inactivation of the X chromosome. Based on the assumption that there is a non-random inactivation of the affected allele, we utilized a popular inactivation assay to test our hypothesis and checked the methylation pattern using HhaI restriction enzyme. The methylation status of the methylation-sensitive enzyme’s restriction sites near the polymorphic CAG repeat in the first exon of the human androgen receptor (AR) locus correlates with X chromosome inactivation. Hence, we analyzed the X-inactivation pattern of blood DNA from the affected patient and her parents. Moreover, the same experiment was performed using the fibroblast DNA, which was only available from the affected individual. The results are shown in Figure 3A. When the genomic DNA from the whole blood samples was amplified without *Hha*I digestion, two polymorphic alleles at the AR locus [AR1 (260) and AR2 (280)] can be seen, as shown in Figure 3A. The assay indicated that the AR1 allele is from the mother and the AR2 allele is from the father. However, after *Hha*I digestion in the blood from the affected girl and the mother, only the AR1 allele could be amplified, indicating the presence of non-random X-inactivation in the patient Figure 3A.

## Discussion

LNS is an inherited neurometabolic disorder and transmitted in an X-linked recessive manner. Based on the inheritance pattern, LNS almost exclusively manifests in male individuals and expresses the classical LND phenotypes. Due to the random inactivation of the X chromosome, heterozygous girls are frequently phenotypically normal. However, rarely, there have been female individuals (n = 14) carrying mutations and showing biochemical and clinical manifestations of LND (Hooft et al., 1968; Hara et al., 1982; Ogasawara et al., 1989; van Bogaert et al., 1992; Yukawa et al., 1992; Yamada et al., 1994; Aral et al., 1996; Yamada et al., 1996; De Gregorio et al., 2000; Jinnah et al., 2000; Inokuchi et al., 2004; De Gregorio et al., 2005; Rinat et al., 2006; Sebesta et al., 2008).

We encountered a female patient from Saudi Arabia showing typical characteristics of the disorder. The 4-year-old girl, throughout her developmental stages, showcased the typical phenotype of LNS, including general developmental delay, self-mutilation, hyperuricemia, hypotonia, delayed speech, spasticity, and seizures (Table 1). Her EEG results revealed a mild non-specific cerebral dysfunction over the left temporal head region. The results of the US revealed a small right kidney for her age and bilateral echogenic foci within the renal collecting system. In our case, the transcription of one of the HPRT1 alleles was blocked due to a *de novo* frameshift mutation (c.539delG) in *HPRT1,* whereas the transcription of the normal allele was inhibited because of the non-random inactivation of the second X-chromosome similar to those of previously published female cases.

A wide range of genetic mutations have been detected across all 14 cases including our case (Figure 3B). Two female cases reported by Aral et al. (1996) and De Gregorio et al. (2000) exhibited non-sense mutations, p.Arg170* and p.Tyr153*, respectively. Only one female patient was identified to carry a stop mutation (Yamada et al., 1996). Two missense mutations (p.Glu14Lys and p.Tyr72Cys) were detected in two different female patients. Interestingly, these patients were diagnosed at a later age compared to other cases. There was only one splice site mutation (c.609+4A>G) reported twice (Jinnah et al., 2000; De Gregorio et al., 2005). A patient carried a translocation severe [46,XX,t(X:2)(q26:p25)] and exhibited by far the most characteristics of LNS reported in the literature (Rinat et al., 2006). There was only one microdeletion of *HPRT1* reported by Ogasawara et al. (1989). The previously reported cases and mutations are listed in Supplementary Table S1.

The classic phenotype of LNS in male individuals includes those of the neurological sequelae, hypotonia, athetoid movements, intellectual disability, dysarthria, and self-mutilation. These are the prominent signs of LNS and are important to differentiate for the correct diagnosis of patients at an early stage. Such features have been reported in many female cases as well. Yet, due to the rare occurrence of the syndrome in female patients, it often goes missed or misdiagnosed (Fu et al., 2014), or it is confused with neurological disorders such as, muscular degenerative disorders, psychological disorders, or several types of cerebral palsy (Hara et al., 1982; Yukawa et al., 1992; De Gregorio et al., 2005). An example of this is in a case of a female patient reported by Hara et al. (1982). The patient was brought to the hospital, owing to her developmental stages with various symptoms and signs that align with the classical phenotype of LNS. Nonetheless, she was only evaluated for LNS at the age of 5 after exhibiting self-mutilation involuntarily leading to the loss of half of her lower lip. It was reported that the girl showed complete deficiency of HPRT1 with a threefold increase in APRT activity, which is a classical characteristic of LNS, and this has been observed in male subjects as well. Enzyme assay is used to confirm the diagnosis of LNS by assessing HPRT1 (2 ± .39 μmol\g Hb\min) and APRT (0.41 ± 0.17 μmol\g Hb\min) enzyme activities. The HPRT1 enzyme activity in erythrocytes is used as a confirmatory test. Moreover, laboratory examinations are used to assess the signs and symptoms of LNS, such as the dramatic high levels of uric acid due to absent purine recycling exceeding the normal values of 208–428 μmol/L in male and 155–357 μmol/L in female individuals.

The decreased level of the HPRT1 enzyme activity in erythrocytes has been detected with a subsequent increase in the APRT enzyme activity in some patients (Ogasawara et al., 1989; van Bogaert et al., 1992; Yukawa et al., 1992; Yamada et al., 1994; Jinnah et al., 2000; De Gregorio et al., 2005; Rinat et al., 2006). On the other hand, almost all cases showed an increase in serum uric acid levels. Similarly, an increased serum uric acid level (403 mmol\l) was observed in our patient. A subsequent consequence of hyperuricemia in LNS patients is kidney micro-injuries and kidney stones (nephrolithiasis). If these conditions go untreated, they can eventually progress into kidney failure. Such consequences have been reported in two female cases: a 29-year-old suffered from nephrolithiasis in one of her kidneys, a high serum uric acid level of 583 µmol\l and true gout, and a two-month-old presented with an advanced level of bilateral nephrolithiasis and acute renal failure.

From reviewing the published cases, it can be said that the most severe characteristics and signs have been seen in two female cases (Hooft et al., 1968; Hara et al., 1982). Both patients had complete deficiency in HPRT1 activity, which could be an indicator of deterioration in their cases. Enzyme deficiency has been associated with psychomotor delays in LNS in male individuals, whereas, in our case, severe dystonia and neurological sequelae, choreoathetosis movements, mild to severe mental deterioration, early onset of psychomotor delay, and psychological impairment were observed.

In conclusion, genetic testing inclusive of X-chromosome inactivation assay coupled with serum analysis and basic clinical manifestations of LNS can be useful for an early and quick differential diagnosis of the disease in female patients. LNS should be considered in female patients exhibiting typical characteristics of LNS, as has been done with male patients.

## Data Availability

The data analyzed in this study is subject to the following licenses/restrictions: The data cannot be publicly released due to patient confidentiality. Requests to access these datasets should be directed to the corresponding authors.
